# B-positive: a randomized controlled trial of a multicomponent positive psychology intervention for euthymic patients with bipolar disorder - study protocol and intervention development

**DOI:** 10.1186/s12888-018-1916-3

**Published:** 2018-10-17

**Authors:** Jannis T. Kraiss, Peter M. ten Klooster, Melissa Chrispijn, Hester R. Trompetter, Anja W.M.M. Stevens, Erica Neutel, Ralph W. Kupka, Ernst T. Bohlmeijer

**Affiliations:** 1 Center for eHealth and Well-being Research, Department of Psychology, Health, and Technology, University of Twente, PO Box 217, Enschede, 7500 AE the Netherlands; 2 Specialized Center for Bipolar Disorders, Dimence group, Pikeursbaan 3, Deventer, 7411 GT the Netherlands; 3 Center of Research on Psychological and Somatic Disorders, TS Social and Behavioral Sciences, Tilburg University, PO Box 90153, Tilburg, 5000 LE the Netherlands; 4 Dutch Association for Manic Depressives and Relatives, Stationsplein 125, Amersfoort, 3818 LE the Netherlands; 5 Department of Psychiatry, VU University Medical Center, Oldenaller 1, Amsterdam, 1081 HJ the Netherlands

**Keywords:** Positive psychology, Well-being, Flourishing, Personal recovery, Bipolar disorder, Intervention, Randomized controlled trial, RCT, Effect, Effectiveness

## Abstract

**Background:**

Bipolar disorder (BD) is characterized by recurrent (hypo)manic and depressive episodes, alternating with euthymic states in which patients are relatively symptom free. Besides clinical recovery, it is important to also strive for improvement of mental well-being and personal recovery. One prominent field focussing on the improvement of well-being is positive psychology. However, studies assessing the effects of positive psychology or personal recovery interventions for people with BD are scarce and have used weak methodological designs. The study described in this protocol article aims to assess the effectiveness of a multicomponent positive psychology intervention (“Living well with bipolar disorder”) adjusted for people with BD in the euthymic phase to improve well-being and personal recovery.

**Method:**

The study concerns a pragmatic randomized multicenter trial. The principle objective of the study is to assess whether the positive psychology intervention offered to BD patients in remission in addition to usual care (CAU) is more effective than CAU. The study will include 112 participants randomized to either the experimental condition receiving the intervention in addition to CAU or the control condition receiving CAU. The study population are patients with BD I or II in the euthymic phase.

The inclusion criteria are 1) diagnosis of BD I or BD II, 2) between the ages of 18–65, 3) four or more supportive sessions in the last year, and 4) only residual depressive or manic symptoms. Patients are excluded if they are in a depressive or manic episode, have current addiction problems or have optimal levels of well-being. Measurements take place at baseline, post-intervention and follow-up 6 and 12 months from baseline. Outcomes of measures include positive well-being, personal recovery, psychopathology, self-compassion, positive relationships, dampening of positive affect and relapse.

**Discussion:**

The outlined study will be the first RCT examining the effects of a multicomponent positive psychology intervention for patients with bipolar disorder. Several limitations, including generalizability of the results and possible attrition issues, are discussed in advance.

**Trial registration:**

This study has been registered in the Netherlands Trial Register (NTR6729) on 12 October 2017.

**Electronic supplementary material:**

The online version of this article (10.1186/s12888-018-1916-3) contains supplementary material, which is available to authorized users.

## Background

Bipolar disorder (BD) is a severe mood disorder and is characterized by recurrent (hypo)manic and depressive episodes, alternating with euthymic phases in which patients are relatively symptom free [1, 2]. BD is subclassified as bipolar I and bipolar II disorder. In the latter, patients solely experience hypomanic episodes but never a full manic episode [3, 4]. Prevalence estimates from the Netherlands reveal a lifetime prevalence of 1.3% and 12-month prevalence of 0.8% for BD I and II [5]. The economic burden in 2009 was estimated at 151 billion dollars per year in the United States [6]. BD is associated with decreased quality of life [7], negative social consequences, such as a disturbed social life and disrupted family interactions [8], issues related to work-performance and productivity [9, 10] and high caregiver burden [11, 12]. Current treatment for euthymic BD patients in the Netherlands includes pharmacotherapy, supportive treatment, psycho education and enhancement of self-management skills, and psychotherapy if indicated [13, 14].

In addition to current symptom-focused treatment, it becomes increasingly important to also focus on personal recovery [15–17]. Leamy, Bird, Le Boutillier, Williams and Slade [18] created a conceptual framework for personal recovery in mental health, containing five processes of personal recovery: connectedness, hope and optimism about the future, identity, meaning in life and empowerment (giving the acronym CHIME) as important factors for personal recovery [18]. Anthony (1993) operationalizes personal recovery in the context of psychopathology and describes it as the ability to live a meaningful, hopeful and contributing life, even in the presence of mental illness [19]. Similarly, Keyes [20] defines mental health recovery as the presence of well-being and not merely the absence of mental illness. Well-being, in turn, includes subjective well-being (i.e. positive affect and life-satisfaction), psychological well-being (i.e. meaning, goals in life, mastery, positive relationships) and social well-being (i.e. contributing to society) [20].

Personal recovery and well-being are particularly important in BD for several reasons. Residual subthreshold symptoms often persist in the interval between mood episodes [21–23]. Moreover, up to 35% of BD patients do not completely recover from a depressive or manic episode [24], which is an important risk factor for relapse [17]. Research also indicates that improvement of well-being protects against the recurrence of mental illness [25–27]. Patients with serious mental illness, such as BD also express dissatisfaction with current primary targets of treatment and instead argue for the importance of personal recovery outcomes [28, 29].

One prominent field of psychology focussing on the improvement of well-being and positive capacities is positive psychology [30]. Positive psychology interventions focus on the enhancement of positive feelings, behaviours, or cognitions and aim to improve well-being [31]. The key processes and goals of positive psychology are similar and central to personal recovery [15, 32] making positive psychology interventions potentially useful for improving both well-being and personal recovery in people with mental illness. The effect of positive psychology interventions has been shown in meta-analyses for both general and clinical populations [31, 33].

To date, however, no study has been conducted assessing the effect of positive psychology interventions for the treatment of people with BD and only a few studies investigated the effect of interventions focussing on the improvement of personal recovery. Deckersbach, Hölzel, Eisner, Stange, Peckham, Dougherty, Rauch, Lazar and Nierenberg [34] report on a small uncontrolled clinical trial with 12 euthymic participants diagnosed with BD using Mindfulness-Based Cognitive Therapy (CBT). Analysis from pre- to follow-up indicated significant moderate to large improvements in outcomes of depressive symptoms (Cohen’s *d* = .75), positive affect and aspects of well-being. Eisner, Eddie, Harley, Jacobo, Nierenberg and Deckersbach [35] conducted a proof-of-concept pilot study with 37 participants with BD who did not have a current major depressive, manic or mixed episode. Significant large improvements were obtained from baseline to post-treatment in psychological well-being, emotion regulation, and emotion reactivity. Finally, Jones, Smith, Mulligan, Lobban, Law, Dunn, Welford, Kelly, Mulligan and Morrison [36] investigated the effectiveness of recovery-focused CBT in a randomized controlled pilot trial (*n* = 67) with care as usual as control. Personal recovery significantly improved from baseline to 6 and 12 months follow-up (*d* = .62) in comparison with a control group receiving treatment as usual. Although no significant effects were obtained in average mood symptoms, patients in the recovery-focused CBT group showed significantly longer time to relapse into depression or mania over a 15-month period compared to patients only receiving care as usual. For example, 32 patients relapsed to either depression or mania (20 CAU v. 12 recovery-focused CBT) and median survival times were longer for recovery-focused CBT (56 weeks) compared to CAU (18 weeks).

In summary, no studies exist investigating the effectiveness of positive psychology interventions for BD patients. Furthermore, studies examining the effect of interventions aimed to enhance personal recovery or well-being for patients with BD are scarce and mostly used underpowered and weak methodological designs. For this reason, we aim to develop, implement and thoroughly investigate the effects of a multicomponent positive psychology intervention for the improvement of well-being and personal recovery for people with BD in an adequately powered randomized controlled trial. This will be the first study specifically evaluating a positive psychology intervention for patients with BD and will use a more sophisticated methodological design than studies before.

The primary objective is to evaluate the effectiveness of an eight-week multicomponent intervention *“*Living well with bipolar disorder*”* added to usual care (CAU) in BD patients in the euthymic phase. Primary outcome is the short and long term improvement of well-being and personal recovery. Secondly, the study aims to investigate whether the intervention in addition to CAU is more effective in improving social participation, and in improving depressive, manic, and anxiety symptoms. Thirdly, we will explore possible working mechanisms for intervention effects, including positive emotions, self-compassion, positive relationships and dampening of positive affect. Fourthly, the study aims to assess whether the intervention combined with CAU is more effective than CAU in reducing recurrence of depressive and (hypo)manic episodes in patients with BD in the long term. Finally, we aim to evaluate the cost-effectiveness of the intervention in addition to CAU for the treatment of euthymic patients with BD compared to CAU.

## Method

### Study design

**Fig. 1:**
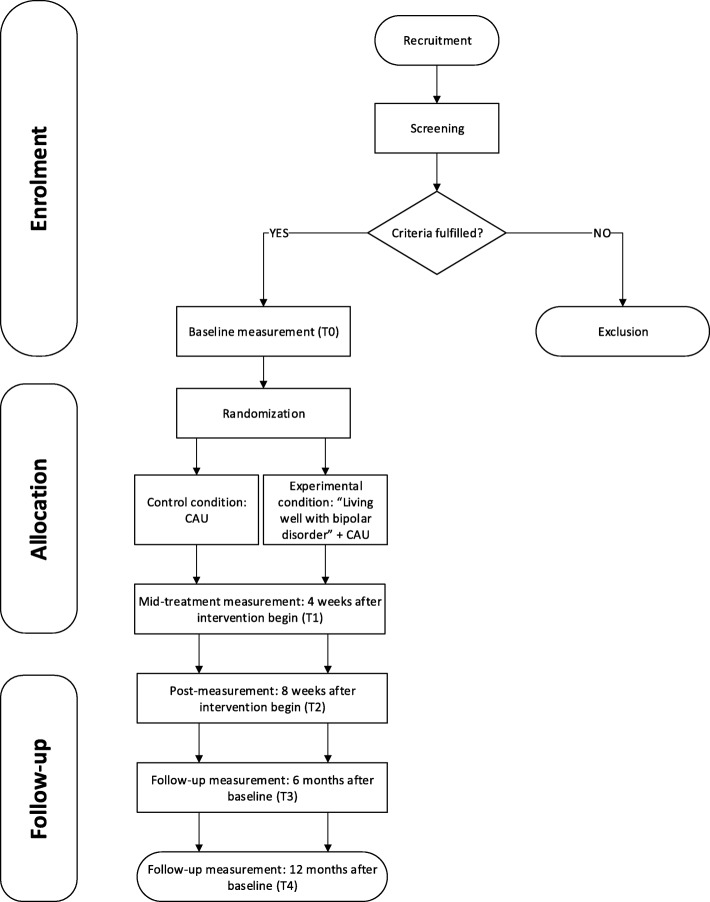
Participant timeline

A pragmatic, parallel-group randomized non-blinded multicenter trial is used to investigate the effectiveness of a multicomponent positive psychology intervention to improve well-being and personal recovery in patients with BD. Since the outcome in BD I and BD II disorder may be different, the sample will be stratified accordingly. Patients in the experimental condition receive “Living well with bipolar disorder” in addition to CAU. Participants in the control condition receive CAU only. Both participants in the control and experimental condition receive CAU according to the Dutch multidisciplinary guideline for BD [13], consisting mainly of self-monitoring of mood and supportive group sessions focusing on functional problems, and psychopharmacotherapy. The study duration is 12 months for each individual and includes five measurement points. Immediately prior to the start of the intervention a baseline measurement is completed (T0), 4 weeks after the start of the intervention a mid-treatment measurement takes place (T1) and immediately following the intervention a post measurement will be conducted (T2). In addition, two follow-up measures will be conducted, 6 months (T3) and 12 months after baseline (T4). Figure 1 shows the intended flow of participants (Additional file 1).

### Participants, eligibility and screening

In order to be eligible to participate in this study, participants must meet the following inclusion criteria: (1) diagnosis of BD I or BD II (assessed using the MINI-international neuropsychiatric interview [37]); (2) between the ages of 18–65; (3) four or more supportive sessions in the previous year with a psychiatrist or psychologist; and (4) presence of residual subsyndromal symptoms. Participants are included if they score between 2 (*minimal symptoms)* and 4 (*moderate symptoms*) for depressive symptoms and between 2 (*minimal symptoms*) and 3 (*mild symptoms*) for manic symptoms on the Clinical Global Impression Scale – Bipolar (CGI-BP). Exclusion criteria are: (1) currently in a depressive or (hypo)manic episode; (2) currently in treatment for addiction problems, or (3) having already optimal levels of well-being. Participants are assumed to have an optimal level of well-being if they score 4 or 5 on at least one item of the emotional well-being subscale together with a score of 4 or 5 on at least 6 of the 11 remaining items of the Mental Health Continuum-Short Form (MHC-SF; [20]).

### Randomization and treatment allocation

Randomization will be centrally conducted by the principal investigator using stratified (by center) block randomisation. For this purpose, randomization lists will be generated beforehand (one list for each participating treatment center) with an online tool (https://sealedenvelope.com/). The lists contain a random sequence of treatment allocations (i.e., participants are either allocated to the intervention or control condition according to the corresponding record) and are divided in blocks of allocations (20 allocations per block). By using blocks of allocations, 20 participants can be allocated to either the intervention or control condition after which the following block is used. This ensures that the group sessions in each center can start as soon as sufficient participants are randomized. The first participant included in the study is allocated according to the first record on the list, the second participant according to the second record on the list and so forth.

### Recruitment

Participants will be recruited from at least four mental health centers with six locations in the east and west of the Netherlands and will start in September 2018. Possible participants are informed by means of flyers and posters distributed in these centers. In order to include sufficient participants, mental health professionals will play an active role, by informing potentially eligible patients about the study and handing out information folders.

### Intervention

We will adapt the positive psychology intervention *“*This is your life*”* [38, 39] for patients with BD in the euthymic phase aiming to enhance personal recovery and well-being. Originally developed as a self-help book, the intervention is primarily based on the well-being theory of Seligman [40] and Ryff’s theory of psychological well-being [41] and comprises different modules focusing on six key components: positive emotions; discovering and using strengths; optimism and hope; self-compassion; resilience and post-traumatic growth and positive relations. Each module contains psycho-education and a range of different positive psychology exercises, such as the “three good things exercise” [33] or the “best possible self” [42]. All modules include proven strategies for improving well-being. In a recent randomized controlled trial, the intervention as guided self-help with email support revealed moderate to large effects on well-being (*d* = 0.66) and effectively reduced subclinical symptoms of anxiety (*d* = 0.63) and depression (*d* = 0.43) in a non-clinical sample [39].

### Intervention adjustments for euthymic BD patients

Since *“*This is your life*”* was not specifically developed for individuals with BD, we decided to customize the intervention content and delivery to the needs of this target group. The adjusted intervention is called “Living well with bipolar disorder”. We changed the mode of treatment delivery from individual self-help to group meetings (8–10 people per group), including eight sessions of 2 h conducted by a specially trained therapist. This setup is believed to give participants the possibility to share experiences with fellow patients and benefit from the presence of other group members.

Each week, a key positive psychology component will be covered and corresponding exercises will be introduced. Participants are also encouraged to keep training with those exercises at home (for 15–30 min per day). All intervention participants receive the self-help book *“*This is your life*”* [43], since several parts of the modules refer to chapters or exercises in the book. The adapted intervention consists of eight different modules covering different topics, such as positive emotions, positive relationships or personal goals. Homework differs depending on the module, but every week participants are encouraged to keep training with the exercises at home. Experiences with the homework is discussed at the beginning of each next session and possible obstacles or other benefits can be shared with the therapist and other group members. In case the module contains a collective exercise, a short debriefing session will take place immediately after the exercise, to discuss experienced benefits or difficulties. Every module contains a short break of 15 min. Every session is finished by a short conclusion in which the therapist summarizes the session and where uncertainties can be broached and questions can be asked.

In spring 2017, a formative user evaluation of *“*This is your life*”* was carried out to evaluate the original intervention contents. Five individuals with BD read and practiced a selection of the original exercises and rated them on a scale from 1 (*not at all*) to 5 (*extremely*), representing the relevance and usability of the exercises. Results of this group were used to further adapt and tailor the intervention to the needs of individuals with BD by taking into account the ratings of exercises, preferences of the patients and also their critical responses.

For several reasons, we decided to put additional emphasis on fostering self-compassion skills by including two sessions of self-compassion. Research on emotion regulation [44–46] emphasizes the importance of disturbed positive emotion regulation (i.e., how an individual reacts to positive emotions) for the onset, maintenance and illness course of BD [47]. Suppression of positive emotions is heightened among BD patients and predicts depressive and also manic symptoms [48, 49]. Additionally, negative thinking about high moods (e.g. “I will lose control if I get excited”) is also elevated in individuals with BD, compared with nonclinical controls [50–52], individuals with remitted unipolar depression [50, 52] and individuals who have had hypomanic experiences, but no psychological disorder [50] and predicts mood symptoms in BD over the period of 1 month [53] and 6 months [49]. Studies show that self-compassion is positively related with adaptive emotion regulation processes, including acceptance and positive reappraisal and negatively correlated with maladaptive strategies, such as thought suppression and rumination [54–56]. An overview of the intervention contents can be found in Table 1.

**Table 1:** Content of “Living well with bipolar disorder” and corresponding example exercises adapted for euthymic BD patients

Module	Contents	Example (home) exercises
1. Introduction & compassion	▪ Participants are welcomed and become familiar with each other ▪ Psychoeducation about personal recovery and compassion ▪ Collective compassion exercise ▪ Homework: Fill in handout about personal goals and optimism	▪ Wish yourself something good: be mindful and identify needs and use your inner voice to repeat your compassionate wish [86]. ▪ Common humanity: Realize that negative feelings and experiences are universal [87, 88].
2. Personal goals & optimism	▪ Based on the handouts, participants talk about personal goals and wishes and specify personal goals in the group ▪ Individually adjusting personal goals ▪ Collective optimism exercise ▪ Reading and discussing letter from an experienced person with BD ▪ Homework: Working on personal goals, writing a letter from the future	▪ Imagine your best possible self: Visualize yourself in a future where everything has turned out in the most optimal way [42, 89]. ▪ Letter from the future: write yourself a letter from a future perspective
3. Positive emotions	▪ Read out letter from the future ▪ Psychoeducation about positive emotions ▪ Collective positive emotions exercises ▪ Homework: Taking a photo of a positive moment or experience, working on personal goals	▪ Three good things: Think about three good things that went well today and savor these moments [90]. ▪ Expressing gratitude: identify what you are grateful for in the context of your illness and share those experiences [91].
4. Coping with fear of relapse	▪ Sharing photos of positive experiences and talking about the photos ▪ Talking about participant’s fears, how fear is experienced and internal barriers ▪ Psychoeducation about fear and (un)healthy emotion regulation strategies ▪ Collective exercises on how to efficiently cope with fear ▪ Homework: Do something you find exciting, perform personal strengths exercise, completing mid-treatment measurement	▪ Learn to tolerate and accept fear as important part of life and learn to regulate positive mood and gain a more open view towards them [43]. ▪ Compassionate coping with inner fear: learn to be compassionate towards yourself, your emotions and your negative experiences [86].
5. Personal strengths	▪ Identifying strengths ▪ Psychoeducation about personal strengths ▪ Personal goals and strengths: which strengths can be used to achieve personal goals? ▪ Homework: Keep training with exercises, record possible benefits and barriers while performing the exercises	▪ Identifying strengths: Describe an activity you enjoy to someone else and he/she names strengths deriving from this activity. ▪ Top 5 strengths: Choose your top 5 strengths that give you energy and pleasure [92, 93].
6. Positive relationships	▪ Participants name skills they gathered in the course of the intervention so far and described one example from the last week ▪ Psychoeducation about positive relationships ▪ Participants describe a relationship they want to reinforce positively ▪ Collective positive relationship exercises ▪ Homework: Keep training with positive relationship exercises	▪ Acts of kindness: Performing unexpected acts of kindness for someone else [94]. ▪ Active-constructive responding: Respond positively to good news shared by someone else. Use active communication skills [95, 96]. ▪ Expressing gratitude [91].
7. Compassion	▪ Psychoeducation about emotional systems and evolutionary background ▪ Collective exercises mindfulness and compassion ▪ Coping with thoughts of inferiority and self-critique ▪ Homework: Writing a response to the letter from the second session, fill in questionnaires for post-measurement	▪ Develop a compassionate inner voice: Write about situation in the past week where you showed self-compassion [86]. ▪ Grandma exercise: Imagine a person you feel comfortable with. Concentrate on how it feels to be together with this person and savor this moment [43].
8. Conclusion	▪ Talking about results of the questionnaires and figuring out which aspects are going well and which should receive some extra attention in the next weeks ▪ Read out response to the letter from the second sessions ▪ Participants thank each other	▪ Not applicable.

### Control group

Participants in the comparison group will receive CAU for BD as described in the Dutch multi-disciplinary guideline for bipolar disorder [13] which comprises supportive sessions with a psychologist or psychiatric nurse and maintenance pharmacological treatment by a psychiatrist. Most patients receive 2–12 supportive sessions per year. CAU includes many psychoeducational elements that have the following aims: to give patients information about the illness in the context of the patients’ life-history, to learn to identify early warning signals and prodromal symptoms, to develop and implement strategies to cope with prodromal symptoms, and to develop plans for acute crisis and stabilizing one’s mood. For some patients, CAU may additionally include psychotherapeutic treatments such as cognitive behavioral therapy and interpersonal therapy. For all patients, current CAU does not primarily focus on personal recovery in terms of emotional and psychological well-being (i.e. meaning, purpose in life, positive relationships).

### Therapists training and treatment manual

Therapists carrying out the intervention will receive a one-day workshop covering the central ideas of *“*Living well with bipolar disorder*”,* including background, goals and possible exercises of the intervention. A treatment manual will be prepared to guarantee a standardized execution of the intervention. The manual includes explanations on each module and also on corresponding exercises. Short session handouts (1–2 pages) will be developed for patients participating in the intervention, explaining and summarizing the topics of each module and will include affiliated exercises and can be used to further exercise at home.

### Study procedure

Initial screening for participation will be performed by therapists working at the treatment centers. To assess symptom severity of possible participants, the Clinical Global Impression Scale – Bipolar (CGI-BP) is used. After possible participants sign the informed consent, the principal investigator contacts the patient and agrees on a time and date to conduct the additional screening. To verify the diagnosis of BD, the MINI international neuropsychiatric interview [37, 57] is used and to assess well-being, participants are asked to complete the Mental Health Continuum – Short Form (MHC-SF) [58]. Eligible participants are then asked to complete the first test battery at baseline. Since the MHC-SF has already been completed for screening reasons before, participants are not asked to complete it again at baseline. Baseline measurements should take approximately 40 min to complete. Afterwards, allocation of participants to the intervention or control group takes place.

The first intervention group will start approximately in fall 2018 and the last group will finish in fall 2019. Four weeks after the intervention begins (T1) and after the intervention has finished (T2), participants in the intervention and control condition are asked to fill out a test battery again. Six (T3) and 12 (T4) months after baseline, participants in the experimental and control group will be asked to complete the follow-up measures. We assume that completing the test battery takes approximately 35 min on average at each measurement point. At T4, participants will be approached for a semi-structured telephone interview with the goal to retrospectively assess relapse into mood episodes in the past 9 months. To perform the interviews, a guideline will be prepared and the interviews will be conducted by student assistants according to a fixed scheme. The trained student assistants are blind to the treatment condition of the participants. One interview will take approximately 30 min.

### Study measures

Almost all data being gathered during the trial are self-reported data that will be collected via an online survey program (https://www.qualtrics.com). In addition, one semi-structured telephone interview will be conducted 12 months after baseline to assess relapse. Participants will be asked to report demographical data including gender, age, marital and employment status, ethnicity and education, as well as the information about the past course of BD, at baseline. The primary outcome is well-being and secondary outcomes include personal recovery, social role participation and symptoms of depression, mania and anxiety. Additionally, processes of positive emotions, self-compassion and positive relationships are assessed and economic outcomes are used to calculate the cost-effectivness. An overview of the study measures at the different time points can be found in Table 2*.*

**Table 2:** Overview of study parameters and measurement points

Questionnaire	Outcome	Screening	T0	T1	T2	T3	T4
			Pre-test	Mid-treatment^a^	Post-test	Follow-up^b^	Follow-up^c^
CGI-BP^d^	Global illness severity	X					
MINI	Diagnosis BD	X					
MHC-SF	Well-being	X	X	X	X	X	X
QPR	Personal recovery		X		X	X	X
s-SRPQ	Social participation		X		X	X	X
QIDS-SR	Depressive symptoms		X		X	X	X
ASRM	Manic symptoms		X		X	X	X
HADS-A	Anxiety symptoms		X		X	X	X
PANAS	Positive emotions		X	X	X		
SCS-SF	Self-compassion		X	X	X		
SPWB	Positive relationships		X	X	X		
RPA	Dampening of positive affect		X	X	X		
Telephone interviews	Relapse						X
EQ-5D-5 L	Quality of Life		X				X
TiC-P	Costs associated with psychiatric illness		X				X
Socio- demographics	Gender, age, education, marital status, living situation, ethnicity		X				

^a^4 weeks after intervention begin

^b^6 months after baseline

^c^12 months after baseline

^d^clinician reported

#### Global illness severity

To screen for the presence of depressive and (hypo)manic symptoms and determine eligibility of possible participants, the Clinical Global Impression – Bipolar (CGI-BP; [59]) scale will be used. This scale comprises three different measures, including severity of depressive and (hypo)manic symptoms, change from preceding phases and change from worst phase of illness. For this study, only the measure assessing the severity of symptoms will be used ranging from 1 (normal, not ill) to 7 (very severely ill). The CGI-BP showed excellent interrater reliability in prior studies [59].

#### Well-being

The Mental Health Continuum – Short Form (MHC-SF) is a comprehensive well-validated measure of well-being [58]. The MHC-SF measures three dimensions of well-being: 1) emotional well-being (three items), defined in terms of the presence of positive feelings, the absence of negative feelings and satisfaction with life; 2) psychological well-being (six items), defined in terms of positive functioning in individual life in terms of e.g. self-acceptance, personal goals, positive relationships, and environmental mastery; 3) social well-being (five items), defined in terms of positive functioning in social life in terms of e.g. social integration and social contribution. Participants rate the frequency of feelings in the last week. A total score can be created by summing all 14 items, where higher scores indicate better positive well-being. The Dutch version of the MHC-SF showed high internal consistency for total scores (*α* = 0.89) and for the subscales *emotional* (*α* = 0.83) and *psychological well-being* (*α* = 0.83) and adequate reliability for the subscale *social well-being* (*α* = 0.74) and correlates well with corresponding aspects of well-being and functioning, showing convergent validity [58].

#### Personal recovery

To comprehensively assess personal recovery, the 15-item version of the Questionnaire about the Process of Recovery is used (QPR; [60, 61]).The scale aims to assess personal recovery in the last 7 days (e.g. “I feel better about myself” or “I can actively engage with life”), with items being scored on a 5-point Likert scale, ranging from 0 to (*disagree strongly*) to 4 (*agree strongly*) and higher scores being indicative of recovery. The internal consistency of the 15-item version has been found to be high (*α* = 0.89) in a sample of psychotic patients [62] and in a group of individuals with a schizophrenia spectrum diagnosis (*α* = 0.93) [61]. For this study, the QPR has been translated into Dutch via forward and backward translation.

#### Social role participation

The Social Role Participation Questionnaire (SRPQ; [63]) assesses social role participation. For this study, the short version of the questionnaire (s-SRPQ; [64]) will be used, which consists of 12 items, measuring the influence of (psychological) health in the past on six social roles (e.g. intimate relationship or employment) along two dimensions: (1) satisfaction with role performance and (2) experienced physical / psychological difficulty. Items are scored on a 5-point Likert Scale, reaching from 0 (*not satisfied at all / no difficulties at all*) to 4 (*very much satisfied / not possible*), with higher scores indicating more satisfaction respectively more experienced difficulties with a social role. The psychometric qualities of the Dutch s-SRPQ were found to be good for both subscales *(α* = 0.86) [64].

#### Depressive symptoms

The self-report version of the Quick Inventory of Depressive Symptomatology (QIDS-SR) [65, 66] assesses depressive symptoms in the past on 16 items. The scale requires individuals to rate different depression symptoms, such as sad mood, concentration, suicidal ideation, general interest, energy/fatigue, sleep, appetite and weight. Items are scored on a 4-point Likert Scale with different answering categories. A total score can be obtained by summing all items, with higher scores indicating more depressive symptomatology. The QIDS-SR has shown to be internally consistent (*α* = 0.86) [65].

#### Manic symptoms

Current manic symptoms are measured using the Altman Self-Rating Mania Scale (ASRM) [67]. The scale consists of five statements that represent different manic symptoms, including feeling happier, self-confident and talkative than normal. All five items are rated on a 5-point Likert scale with different answering categories. A total score can be obtained by summing all items, with higher scores indicating more manic symptomatology. The ASRM has high test-retest reliability [67], has been shown to be sensitive to changes of clinical states [68] and to predict related measures in non-clinical student samples [69].

#### Anxiety symptoms

The anxiety subscale of the Hospital Anxiety and Depression Scale (HADS-A; [70]) is used to assess anxious symptomatology. The HADS-A aims to measure anxiety symptoms in 7 items. Participants rate the frequency of symptoms (e.g. “Worrying thoughts go through my mind”) on a scale ranging from 0 (“not at all”) to 3 (“very often”) and higher scores indicate higher anxiety symptoms. The Dutch version of the HADS-A [71] has been shown good internal consistency in a sample from the general population (*α* = 0.84) and in a sample of psychiatric outpatients (*α* = 0.81).

#### Positive emotions

The Positive and Negative Affect Schedule (PANAS) [72] measures current emotions on two different dimensions: (1) positive and (2) negative affect and includes 20 items describing emotional states (e.g. “*active*” or “*anxious*”). Participants can score those states on a 5-point Likert scale, representing the extent to which they experience an affect at this moment or have experienced in the past week, reaching from 1 (*very slightly or not at all*) to 5 (*extremely*). The scores can be summed up to gain scores for positive and negative affect respectively, with higher scores indicating higher affectivity. For this study, the Dutch version of the PANAS and only the positive affect subscale will be used, which showed acceptable reliability (*α* = .79) [73].

#### Self-compassion

The Self-Compassion Scale – Short Form (SCS-SF) [74, 75] measures the process of self-compassion on six dimensions: (1) self-kindness, (2) self-judgment, (3) common humanity, (4) isolation, (5) mindfulness and (6) over-identification and contains 12 items (e.g., “*When I fail at something important to me I become consumed by feelings of inadequacy”*). Each dimension is assessed by two items, which are scored on a 7-point response scale ranging from 1 (*almost never*) to 7 (*almost always*), representing the extent to which an individual experiences certain aspects of self-compassion. Higher scores indicate an increased degree of self-compassion. Reliability of the total Dutch SCS-SF was shown to be good (*α* = .87). Solely total scores of the SCS-SF will be used for further analyses, since the psychometric properties of the subscales were questionable [74].

#### Positive relationships

The process of positive relationships is assessed using the Scales of Psychological Well-Being (SPWB; [76]), which assesses psychological well-being on six different dimensions (e.g. environmental mastery, self-acceptance). For this study, the subscale *positive relations* will be used measuring the extent to which an individual experiences meaningful intrapersonal relationships with other people (e.g. “*People would describe me as a giving person, willing to share my time with others*”). Items are scored on a scale ranging from 1 (*strongly disagree*) to 6 (*strongly agree*) with higher scores indicating more positive relations with others. Different versions of the SPWB exist within literature, differing in number of items per subscale (reaching from 3 items to 20 items per subscale). For feasibility reasons and since the short version of this subscale (3-items) showed unacceptable internal consistency (*α* = .44–.52), we decided to use the 9-item version of the *positive relationship* subscale, which showed acceptable internal consistency in two previous studies (*α* = .77) in samples of psychology students and professionals from a divers occupation background [77].

#### Dampening of positive affect

To assess the process of dampening the Responses to Positive Affect Questionnaire (RPA; [78, 79]) is used, which consists of 17 items and measures cognitive responses to positive affective states. Respondents rate the items on a 4-point Likert scale, ranging from 1 (*almost never*) to 4 (*almost always*). For this study, only the subscale *dampening* is used (e.g., “I don’t deserve this”), which assesses the tendency to cognitively avoid or suppress positive emotions (eight items). Scores of the scales are calculated by summing up the scores on the items. The Dutch version, which is used in this study, showed satisfactory internal consistency (*α* = 0.80) for the dampening subscale [78].

#### Relapse

Semi-structured telephone interviews will be performed with people of both the intervention and control group. Goal of the interviews is to retrospectively illustrate the mood development in the time after the intervention and to capture depressive or manic mood swings with the Life Chart Method [80]. The interviews allow to graphically score severity of mood swings, the time they appeared (i.e., in which month) and which type of mood swings appeared (e.g., rapid cycling). The interview has been applied successfully in a previous study to measure relapse [14].

### Economic measures

#### Quality of life

The EQ-5D-5 L [81] is a quality of life measure consisting of five items representing five dimensions (mobility, self-care, usual activities, pain/discomfort and anxiety/ depression). For each dimension/item, individuals rate the extent of problems ranging from ‘no problems’ to ‘extreme problems’.

#### Costs associated with psychiatric illness

The Trimbos and iMTA questionnaire on costs associated with psychiatric illness (TiC-P) [82] is a measure of health care utilization and production loss in patients with psychiatric disorders. Items are generic and not related to a specific psychiatric disease. A first part of the TiC-P includes 9 structured no/yes items on medical consumption (e.g. contact with specific mental health care providers). A second part (13 items) consists of the Short Form-Health and Labour Questionnaire, a generic instrument to collect data on productivity losses due to health problems (e.g. absence from work).

### Data collection, management and storage

Data will be handled confidentially in accordance with the Dutch Personal Data Protection Act. For the purpose of this study, a data management plan has been created with DMPonline (https://dmponline.dcc.ac.uk). Details of data management procedures can be requested from the first author of this manuscript. Quality checks, including double data entry and range check for data values will be performed by the first author (JK) and an additional researcher. Personal data will be coded with an individual ID-code, which is not relatable to the participant. All collected data will be stored in a file containing only the identification code. The coded research data will be stored at the BMS Datalab of the University of Twente for a period of 15 years. In this time period, data is accessible to other researchers. After the period of 15 years, data will be stored in long time storage at Data Archiving and Networked Service by the Royal Dutch Academy of Sciences. Participants who want to be informed about their personal data or who want their data deleted can send a request to the principal investigator.

### Study integrity

The study protocol has been designed in accordance with the SPIRIT STATEMENT [83] and the study has been approved by the Medical Ethical Committee Twente (Proposal No: NL62997.044.17). The study will be carried out according to the principles of the Declaration of Helsinki (64th WMA General Assembly, Fortaleza, Brazil, October 2013) and the Medical Research Involving Human Subjects Act (WMO).

### Statistical methods

#### Power calculation

The sample size calculation for this trial is conservatively based on the ability to detect at least a moderate effect of Cohen’s *d* = 0.60 in the post-hoc tests on the primary outcome (well-being) at post-intervention (T1). For a two-sided independent t-test with 80% power and *α* = 0.05, this requires 45 patients for both treatment groups. Taking a maximum drop-out rate of 20% into account, a total of 112 patients will need to be included for the per-protocol analysis.

#### Statistical analyses

Analyses will be done on both intention to treat (ITT) and per-protocol basis. The primary ITT analyses will be performed using linear mixed modelling (LMM) that adequately deals with missing at random data and the nested structure of repeated-measures data. LMMs with time, treatment and time-by-treatment interactions will be performed to test the effectiveness of the intervention in improving continuous primary and secondary outcomes of well-being, personal recovery, psychopathology, sef-compassion, positive relationships and dampening. Post-hoc independent t-tests with Holm-Bonferroni correction will be performed to test for significant between-group differences at all time-points. Based on estimated marginal means and corresponding standard errors from the LMM models, between-group standardized effect sizes will additionally be expressed as Cohen’s d with 95% confidence intervals (CI). Binary relapse data from the interviews will be analyzed with Kaplan Meyer survival estimates to compare the time to relapse and relapse rates between the intervention and control group. Differences in the proportion of relapsed patients and predictors of relapse will be additionally examined using generalized (binary) LMMs with post-hoc chi-square tests and relative risks (RR) with 95% CI to examine the significance and magnitude of differences at each follow-up point. Moderation and mediation analyses will be conducted to explore possible working mechanisms of the interventions. To calculate the cost-effectiveness of the intervention, quality adjusted life years (QALYs) will be taken into account as primary utility measure. QALYs will be calculated from the EQ-5D-5 L. The incremental cost-utility ratio (ICUR) will be calculated by dividing the difference in costs calculated from the TiC-P by the difference in the QALYs produced by the two groups. The ICUR is expressed as costs per QALY gained.

## Discussion

The presented study aims to examine the short- and long term effectiveness of the multicomponent positive psychology intervention *“*Living well with bipolar disorder*”* for euthymic BD patients to enhance personal recovery and well-being. In the present article, we described the intervention development process and several adaptations made to the original program to tailor the intervention for the target group. The study will be conducted in at least four different outpatient treatment centers in the Netherlands, all specialized in the treatment of BD. The intervention group will receive the intervention program in addition to CAU and the control group will receive CAU only.

Several potential limitations of the study have to be considered in advance. Firstly, since participants in this intervention are euthymic (i.e. not in a syndromal depressive, hypomanic or manic episode), results of the trial cannot be generalized to the entire patient group in all phases of BD. Secondly, the study will not be able to determine which specific elements of the interventions lead to possible effects of the intervention. Non-specific factors (e.g. social contact during group lessons or placebo effect) can thus not be ruled out as possible confounding variables. However, since we mainly aim to investigate the effect of the intervention in real-life clinical practice and our study concerns a pragmatic randomized trial, this limitation is not considered to impair the value of our study [84]. Further research could investigate the effectiveness of the intervention to determine whether the intervention is equally effective when taking non-specific factors into account. Thirdly, non-adherence to the intervention or dropout from the assessment might occur. Both types of attrition are likely to bias the results [85].

Our study will be the first to explore the effectiveness of a positive psychology intervention for the treatment of people with BD. In addition, it will be the first study to be implemented that focusses on the improvement of primarily personal recovery and well-being and uses a more sound design than existing pilot studies. The results of the study are expected to broaden the evidence base of clinical positive psychology and personal recovery and potentially generate novel treatment methods for people suffering from BD.

## Additional file

Additional file 1: Participant timeline. Participant activity throughout the trial. (PDF 122 kb)

## Abbreviations

ASRM: Altman self-rating mania scale
BD: Bipolar disorder
CAU: Care as usual
CI: Confidence intervals
GLMM: Generalized linear mixed modelling
HADS-A: Hospital anxiety and depression scale - anxiety
ITT: Intention-to-treat
MHC-SF: Mental health continuum – Short form
PANAS: Positive and negative affect schedule
QALYs: Quality adjusted life years
QIDS-SR: Quick inventory of depressive symptomatology – Self-report
QPR: Questionnaire about the process of recovery
RPA: Responses to positive affect questionnaire
RR: Relative risks
SCS-SF: Self-compassion scale – Short form
SPWB: Scales of psychological wellbeing
SRPQ: Social Role participation questionnaire
