# Supplementary figures and images for: B-positive: a randomized controlled trial of a multicomponent positive psychology intervention for euthymic patients with bipolar disorder - study protocol and intervention development
Source: BMC Psychiatry. 2018 Oct 17;18:335. doi: 10.1186/s12888-018-1916-3 (PMC6192172; doi:10.1186/s12888-018-1916-3)

## Enrolment

## Allocation

## Follow-up

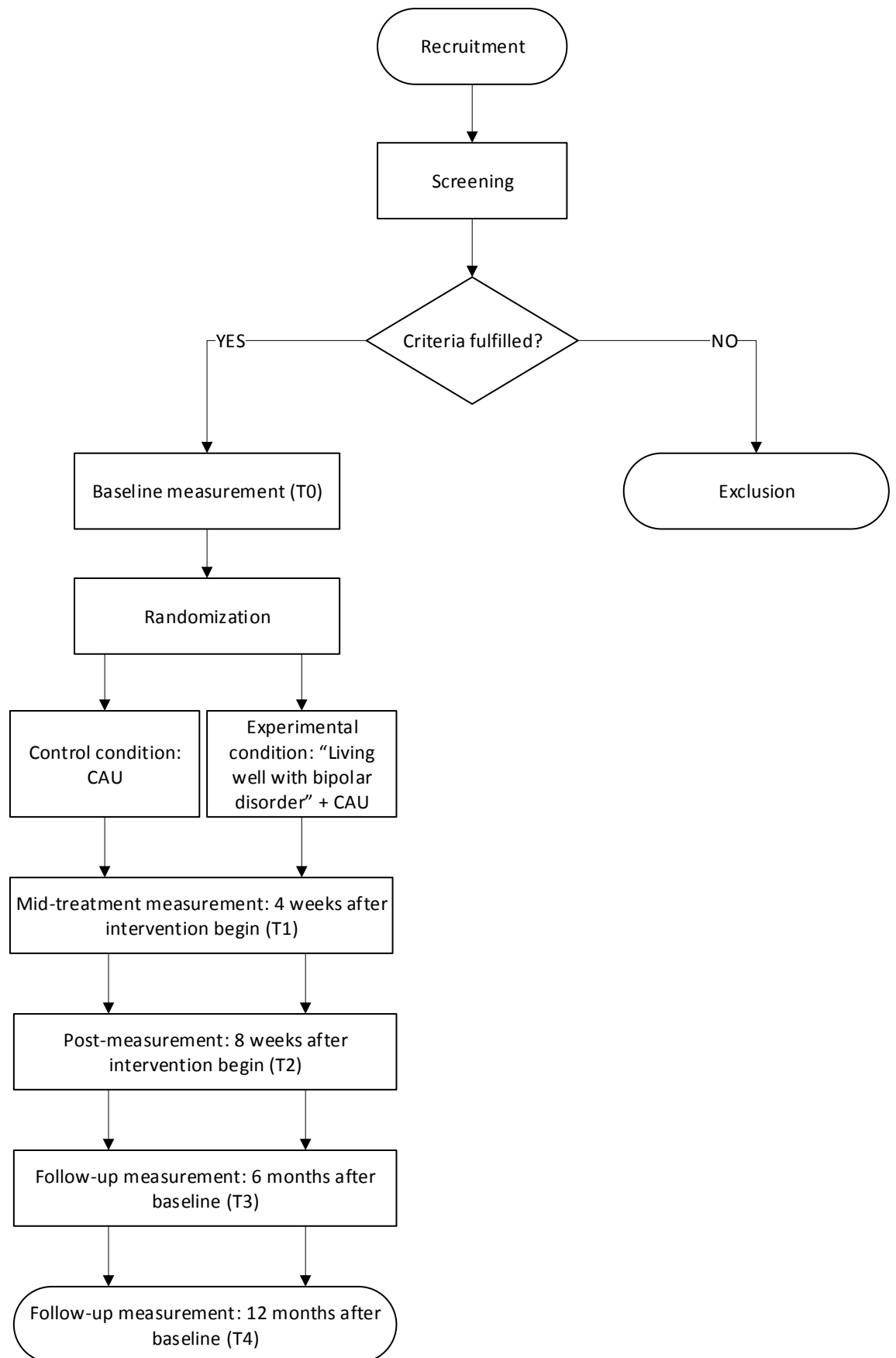

Supplement: Supplementary file 1 — Participant timeline. Participant activity throughout the trial. (PDF 122 kb) [file 12888_2018_1916_MOESM1_ESM.pdf]
